# Supplementary material for: Proteomic analysis shows decreased type I fibers and ectopic fat accumulation in skeletal muscle from women with PCOS
Source: eLife. 2024 Jan 5;12:RP87592. doi: 10.7554/eLife.87592 (PMC10945439; doi:10.7554/eLife.87592)
Supplement: Figure 4—source data 1. [file elife-87592-fig4-data1.zip › Figure 4-Source data 1.pptx]

## Slide 1
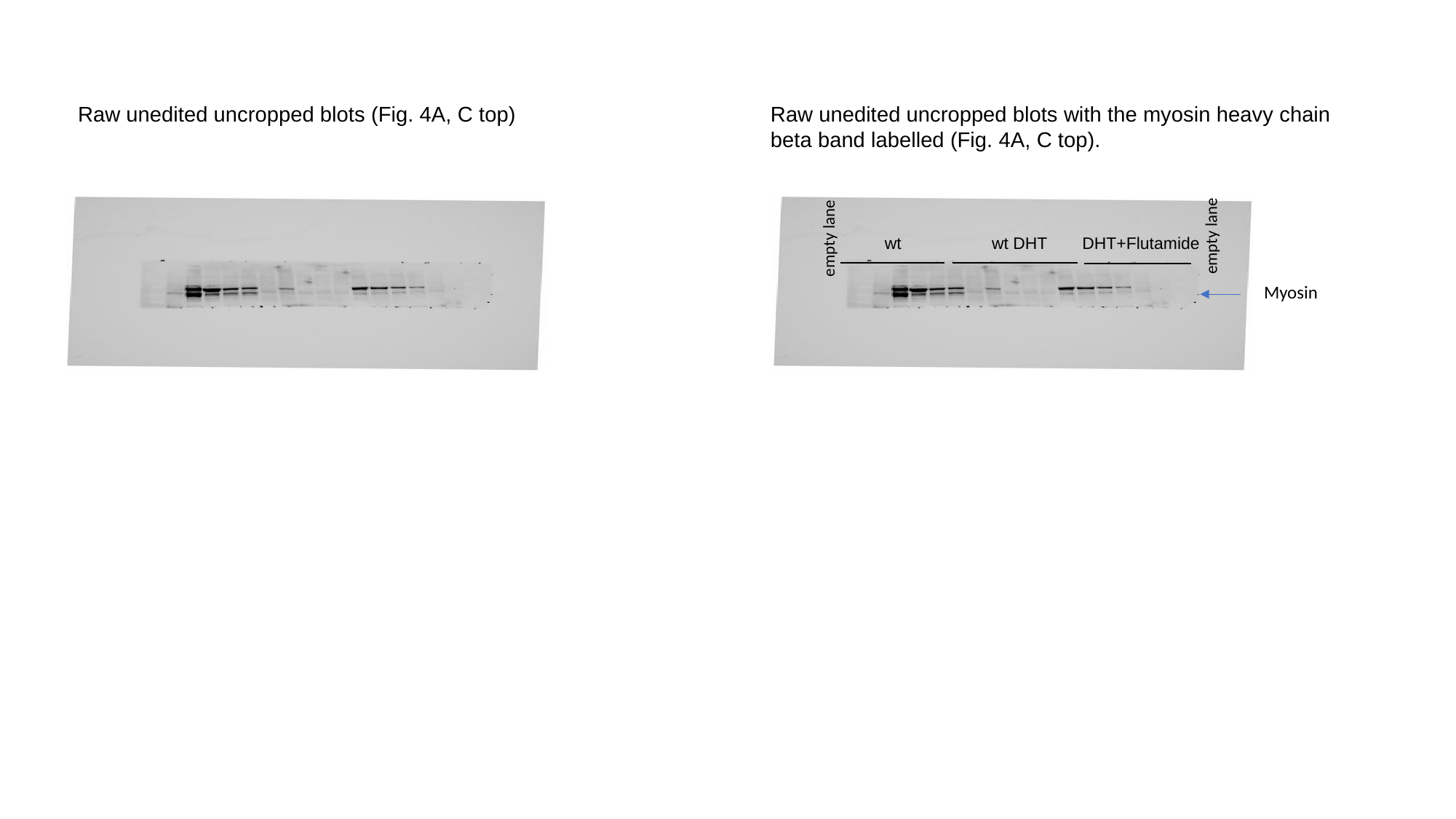

Raw unedited uncropped blots (Fig. 4A, C top)
Raw unedited uncropped blots with the myosin heavy chain beta band labelled (Fig. 4A, C top).
empty lane
empty lane
wt
wt DHT
DHT+Flutamide
Myosin

## Slide 2
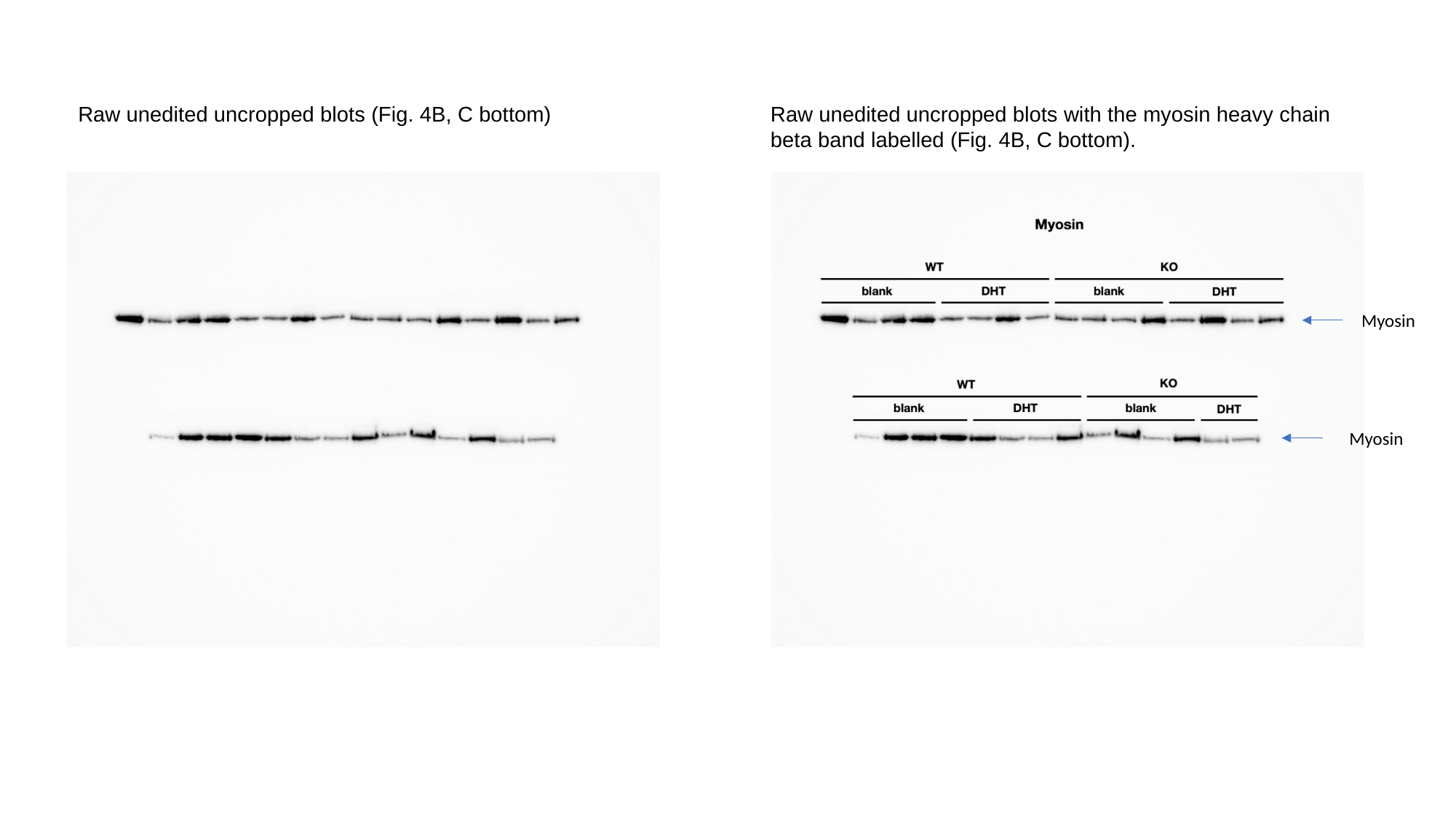

Raw unedited uncropped blots (Fig. 4B, C bottom)
Raw unedited uncropped blots with the myosin heavy chain beta band labelled (Fig. 4B, C bottom).
Myosin
Myosin
