# Supplementary figures and images for: Proteomic analysis shows decreased type I fibers and ectopic fat accumulation in skeletal muscle from women with PCOS
Source: eLife. 2024 Jan 5;12:RP87592. doi: 10.7554/eLife.87592 (PMC10945439; doi:10.7554/eLife.87592)

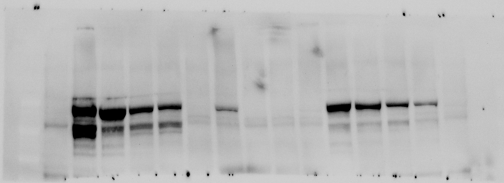

Supplement: Figure 4—source data 1. [file elife-87592-fig4-data1.zip › Full unedited 2.png]

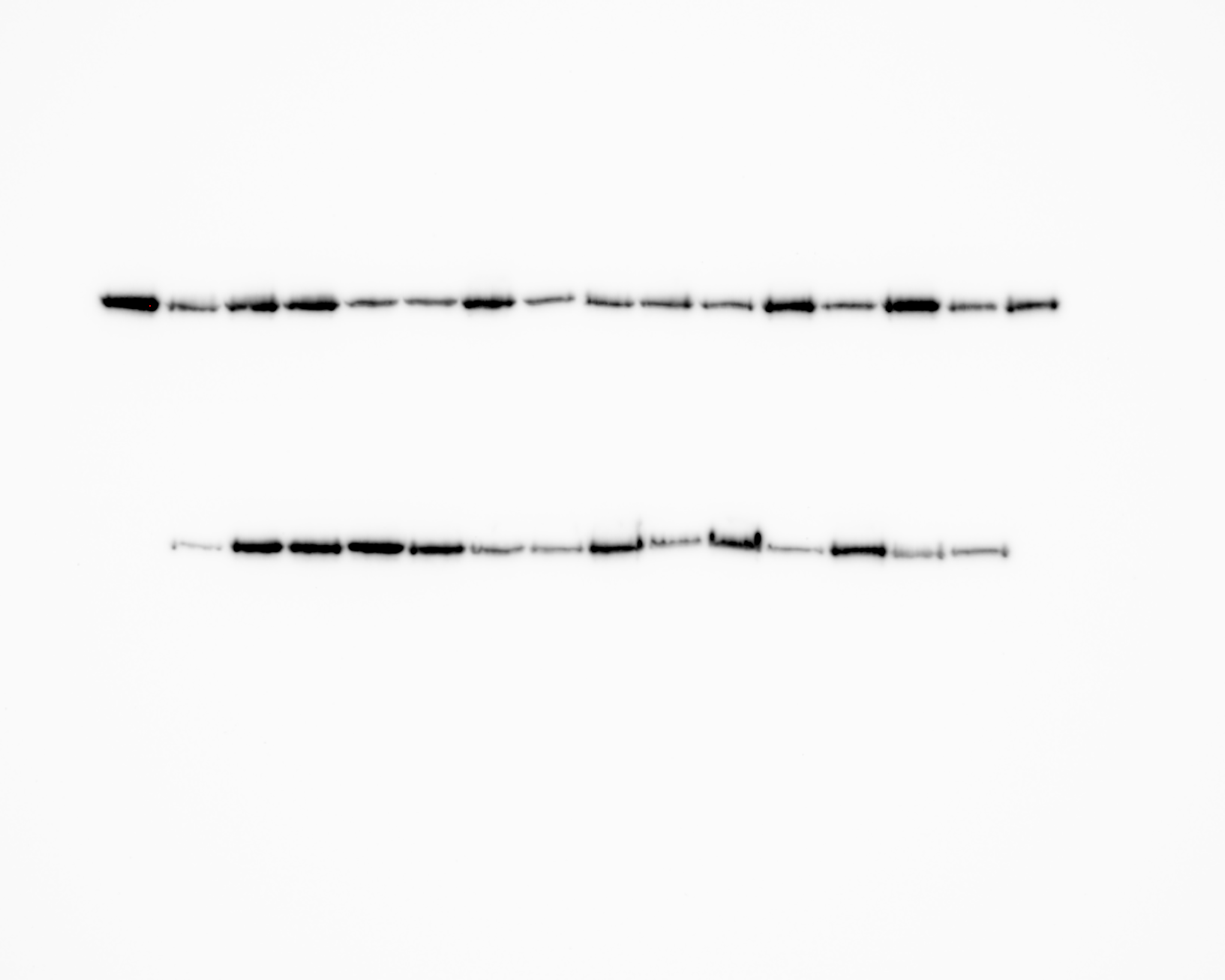

Supplement: Figure 4—source data 1. [file elife-87592-fig4-data1.zip › Full Unedited.tif]
